# The Ethical Basis for Promoting Nutritional Health in Public Schools in the United States

**Published:** 2011-08-15

**Authors:** Patricia B. Crawford, Wendi Gosliner, Harvey Kayman

**Affiliations:** School of Public Health, University of California, Berkeley, CA 94720-3104; University of California, Berkeley, California; School of Public Health, University of California, Berkeley, California

## Abstract

Schools may have an ethical obligation to act in response to the precipitous increase in the incidence of obesity among children. Using a bioethics framework, we present a rationale for school programs to improve the nutritional quality of students' diets. Because children are required to spend half their waking hours in school and because they consume a substantial portion of their daily food there, school is a logical focus for efforts to encourage healthy dietary behaviors to prevent obesity and its consequent individual and collective costs. We suggest that beyond strategic considerations, the concept of the common good justifies actions that may appear to conflict with freedom of choice of children, parents, and school staff, or with the interests of food and beverage companies.

## Introduction

Public schools have an obligation to question and refute policies that do not benefit their students and their communities and a corresponding responsibility to protect students, for whom school attendance is mandated, from harm. However, implementation of change in school procedures and policies presents challenges and requires an ethical justification for the change and feasible methods for accomplishing it.

The mission of schools is broader than simply teaching academic skills. Schools have long accepted responsibility for supporting the health of their students, for example, by requiring immunizations, providing health screenings, and by offering meal programs that support their students' nutritional health. Nutritional health is associated with academic performance (1), and well-nourished students are better able to learn and less likely to miss school for health reasons (2). Research shows that children from low-income families who participate in school breakfast programs score higher on standardized tests and have better school attendance than similar students who do not participate (3). Breakfast programs also improve classroom behavior and attentiveness (4). In 1904, Robert Hunter wrote, "It is utter folly, from the point of view of learning, to have a compulsory school law which compels children, in that weak physical and mental state . . . to sit at their desks, day in and day out for several years, learning little or nothing . . . because hungry stomachs and languid bodies and thin blood are not able to feed the brain" (5).

Focusing on nutritional health promotion in schools can support the common good by reducing the impact, including substantial financial costs, of future diet-related disease associated with the childhood obesity epidemic. Furthermore, optimizing nutrition in childhood is critical to learning and future productivity. We must consider whether schools have an ethical obligation to serve the common good in this area, even if the actions they take appear to conflict with the autonomy or freedom of choice of children, parents, and school staff, or the interests of food and beverage companies.

The purpose of this article is to present a bioethics framework for justifying stricter regulation of school food, specifically, to determine whether this type of health promotion in schools is ethically justified (6). To determine whether current school environments meet an ethical threshold or whether these environments fall short and should be altered, we will apply Beauchamp and Childress's 4 foundational principles for a discourse on the ethics of a biomedical intervention: autonomy (addressing conflict around individualism), beneficence (addressing the social benefit), nonmaleficence (addressing the issue of doing no harm), and justice (addressing equity in burdens and benefits) (7). We describe the underlying problem of rapidly increasing incidence rates of childhood obesity and the potential role of schools in altering the trend.

## Schools' Roles in Addressing Childhood Obesity

The National School Lunch Program was established in 1946 to "safeguard the health and well-being of the Nation's children" as a "measure of national security" by preventing the widespread malnutrition that disqualified many military recruits during World War II (8). Early program participants were served balanced meals to ensure consumption of vegetables, protein, starches, and dairy products according to the best nutrition standards of the time (5). Participation in the program, which was expanded during the 1960s and 1970s, was demonstrated to improve children's diets (9).

### The obesity epidemic

With the tripling in obesity rates among children (10), schools face new challenges. Approximately 1 in 3 children born in 2000 will develop diabetes in his or her lifetime (11), and in a large study of children aged 5 to 17 years, 39% of those who were obese had 2 or more risk factors for cardiovascular disease (12). Poppendieck, in advising policy makers on the benefits of putting money into healthy school foods today to reduce future health care expenditures, calls her recommendations "Pay now or pay later" (13).

### Children's inadequate nutrition

Although children today are consuming sufficient or even excessive food calories, they are not meeting the nutritional requirements described in the federal government's Dietary Guidelines for Americans (14).  Children's intake of fruits, vegetables, and whole grains does not come even close to current recommendations. Furthermore, children aged 5 to 18 years consume approximately 720 to 950 empty discretionary calories per day (15). Calories from added fats and sugars are displacing those from the nutrient-rich foods needed for growth and health.

### Schools' provision of food to students

Children spend up to half their waking hours in school, where they may consume as much as one-third to one-half of their daily calories. Therefore, the school food environment is a logical focus for efforts to encourage healthy dietary behaviors. Today, school food service includes 2 competing arms — the federally regulated reimbursable National School Lunch and School Breakfast programs (8,16) and the competitive foods marketplace, which has expanded substantially during recent decades. Competitive foods and beverages are those foods sold throughout schools in vending machines, school stores, snack bars, and at fund-raisers. These are typically foods of low nutritional quality, including sweetened beverages, chips and other salty snacks, and sweets such as cookies and pastries (17,18).

During a typical day in the first 5 years of the 21st century, 55% of high school students and 44% of middle school students consumed competitive foods at school, frequently instead of school meals (19). Although states and school districts can voluntarily impose restrictions on competitive foods, these agencies are often unaware of the impact of the school food environment on student health. This lack of awareness, coupled with the funding that competitive foods provide to schools, has led to prolonged inaction. However, data now indicate that reductions in competitive food offerings can actually increase meal program participation rates, thereby increasing food service department revenues rather than reducing them as is often feared by school administrators (20). Efforts to promote and increase access to the meal program can be key to school-based efforts to reduce obesity, benefiting both children and schools.

### Recent trends in school food regulation

California in 2005 became the first state to legislate statewide nutrition standards to regulate sale of competitive foods and beverages in grades kindergarten through 12 (21). Evaluation studies of California's implementation of the legislation reveal that schools were successful at eliminating or severely reducing offerings of noncompliant (less nutritious) competitive foods and beverages in schools (20,22). The food and beverage industry replaced or adapted snack foods to meet the new guidelines mandated by the legislation. For example, sports drinks replaced sodas, baked chips replaced original varieties of chips, and reduced-fat crackers replaced original crackers. Although the new offerings met the letter of the legislation's requirements to limit fat, sugar, and calories, they did not substantially increase the availability of such health-promoting foods as fruits, vegetables, whole grains, and low-fat dairy foods.

During the past decade, other states and municipal governments have implemented new obesity-prevention policies in schools with respect to competitive food sales. These policies vary considerably by state and locality. Although competitive foods continued to be available in most schools in the latter half of this decade (19), more than half of all states and several local authorities adopted policies regarding such foods that were more restrictive than those mandated by US Department of Agriculture (USDA) regulations (15).

In recent years, perhaps in response to the variability of state and local requirements, Congress and USDA have been pressured to revisit the issue of school food quality. Furthermore, 2 Institute of Medicine (IOM) reports (15,23) recommended improvements to both competitive and school meal food offerings on the basis of the strongest scientific evidence available. In December 2010, the Healthy, Hunger-Free Kids Act of 2010 was signed into law (24). The act requires the Secretary of Agriculture to establish science-based nutrition standards within a year of enactment. The standards apply to all foods and beverages served outside school breakfast or lunch programs anywhere on school campuses. The extent to which these standards will fully meet the Institute of Medicine recommendations is not clear.

Furthermore, the Healthy, Hunger-Free Kids Act mandates significant improvements to the National School Lunch and School Breakfast programs whereby school meals will be aligned with dietary recommendations for children as outlined in federal Dietary Guidelines for Americans. As the new standards are implemented, school meals will provide increased offerings of nutritious items (eg, fruits, vegetables, whole grains, 1% or nonfat milk) and decreased offerings of foods high in fat, sugar, and sodium. Meal reimbursement rates will be increased slightly to support the purchase of the nutritious offerings. If these policies are implemented as recommended by IOM, significant improvements in nutrition will be realized. However, the political challenge of effectively limiting the sale of less nutritious foods and the economic challenge of paying for more healthful options could result in more limited improvements than are needed to enable schools to promote children's optimal nutritional health.

## Application of a Bioethics Framework for Change in School Nutrition

Opponents of school food regulation argue that people have the right to choose the foods they eat. However, we structure and regulate many student activities in the school setting and do not consider doing so an abridgement of children's rights. The argument that a child has the right to choose foods of poor nutritional quality at school conflicts with the societal value of child protection. A child's right to freedom from obesity is among the 54 binding standards and obligations of the 1989 United Nations Convention on the Rights of the Child (25).

Beauchamp and Childress's (4) 4 foundational principles of biomedical ethics — autonomy, beneficence, nonmaleficence, and justice — can help address the question of whether a mandate to provide nutritious foods to children at school meets bioethics standards that justify regulatory action.

1. **Autonomy.** The conflict between school nutrition interventions and individual rights can be summarized as follows: who is responsible for ensuring that a child eats healthful foods — the parent, the child, or the school? Children are not autonomous agents at home or at school. Because children do not have the knowledge and experience needed to choose foods on the basis of nutritional quality, responsible parents provide foods in the home from which the child can reasonably select. For example, parents would rarely serve candy alongside vegetables on the dinner table and expect their children to choose the vegetables instead of the candy. Similarly, school authorities are responsible for offering foods from which the child can select but limiting choices to those that provide nutritional benefit rather than harm.
2. **Beneficence.** Core to the mission of the National School Lunch and School Breakfast programs is provision of foods that meet the recommended dietary guidelines for optimal nutrition for children. However, efforts to encourage children to eat nutritionally sound school meals are undermined by provision of snacks and beverages that compete with healthier meals. Even partially regulated snack foods compete with healthier meals. Offering nutritious, appealing foods at school meals without competition from less healthy snack foods optimizes students' opportunity to consume a health-promoting diet. Furthermore, the provision of a healthful school meal can serve as a model for educating children and parents alike. From its origins, the National School Lunch Program has asserted: "The educational features of a properly chosen diet served at school should not be under-emphasized. Not only is the child taught what a good diet consists of, but his parents and family likewise are indirectly instructed" (8).
3. **Nonmaleficence.** The principle of nonmaleficence is based on the premise that an intervention should not inflict harm. Providing nutritional foods does not cause harm. However, providing easy access to foods of poor nutritional quality should be construed as causing harm. The ongoing situation in which schools and their suppliers profit from sales of unhealthy foods may be purposely disguised by efforts to teach children not to choose the less healthy food that is offered. Observers might argue that children will not eat foods that are held to a higher nutritional standard; however, children will not go hungry. Students at schools providing nutritious food offerings will still have access to a variety of food choices along with the option of bringing foods from home. Furthermore, students have reported a preference for healthy, fresh food choices over other snack foods (26).
4. **Justice.** The principle of social justice demands that humans be treated fairly, with an equitable distribution of benefits and burdens. Distributive justice takes differences into account and recommends that social and economic inequities are acceptable if they are consistent with just principles and lead to the greatest benefit for the least advantaged (27). Schools provide an opportunity to address social inequities so that children from disadvantaged families have an equal opportunity to become productive citizens. Marketing of foods and beverages on school grounds (eg, in classroom materials, on sporting equipment, by signage) is a school fund-raising technique. Frequent exposure to this marketing in schools in low-income areas where children are at greater nutritional risk is at odds with fairness and social justice. Children from low-income families often experience more psychosocial stresses; having access to healthful foods may modify the effects of these stresses on children's growth and development. Although access to healthful food in schools will be of the greatest benefit to those with the fewest resources, all children benefit from improved nutrition. Doing harm, especially to the most vulnerable children, can never be justified. Selling foods of poor nutritional quality for profit, even if for support of desirable sports or music programs, is an example of such harm.

## The Ethical Basis for Future Action

Providing foods of poor nutritional quality to finance school programs and profit commercial entities fails to meet society's ethical obligation to minimize harm, provide benefit, and protect vulnerable children who are a captive audience. Children are suffering as a consequence of such practices, and children from low-income families, who are most vulnerable to food insecurity, are at greatest risk for damage from consuming empty calories at school. Fostering optimal nutrition not only protects against obesity but is also essential for maximizing cognitive function and academic performance (2). Although new school policies related to health education, school food offerings, and physical education often have been well-received, they rarely have been of sufficient strength to produce demonstrated changes in child obesity rates. Using a bioethics framework, we can begin to formulate a rationale for interventions that support the crucial role schools play in providing nutritious, appealing meals that help children meet their dietary requirements. Schools can and should model an environment that promotes learning and health. In that context, interventions should limit competitive foods to only those foods that contribute to meeting the Dietary Guidelines for Americans and do not contribute empty calories. Only foods that support children's nutritional health should be offered at public schools, and available competitive foods should be equally healthy supplements to the school meal, not less healthy alternatives. These interventions are in children's best interests.

The precautionary principle (28) states, "When an activity raises threats of harm to human health or the environment, precautionary measures should be taken even if some cause and effect relationships are not fully established scientifically." The principle implies that society has a responsibility to intervene and protect the public from exposure to harm where scientific investigation identifies a plausible risk. The risk that malnutrition poses to children's ability to learn is well-documented (1,2). Recent studies link provision of improved nutrition and physical activity at school to improved academic performance for students, especially among low-income minority students (29,30). Providing a healthy diet would help minimize disparities in learning, and children whose families are least able to provide consistent access to adequate food would benefit most substantively. The risk that poor nutrition and obesity pose to children's future health (eg, osteoporosis, heart disease, diabetes) is also well-documented. There is no justification for the promotion of diets that increase those risks. Societal will is needed to provide the required resources to help children achieve nutritional health and simultaneously develop healthy lifetime eating habits. The best interests of children and society demand no less.
